# A new type of silica-induced “moundless” pitting corrosion in copper observed in Japan

**DOI:** 10.1016/j.heliyon.2022.e10110

**Published:** 2022-08-05

**Authors:** Masahiro Sakai

**Affiliations:** College of Design and Manufacturing Technology, Muroran Institute of Technology, 27-1 Mizumoto, Muroran, Hokkaido, Japan

**Keywords:** Copper, Pitting corrosion, Freshwater, Verdigris, Silica

## Abstract

A new type of pitting corrosion in copper, namely “moundless” corrosion, has recently been reported in Japan. This type of pitting corrosion has some unique morphological features that differ from ordinary types of pitting corrosion, such as type I or type II. Firstly, this type of pitting corrosion has no mound of corrosion products that cover the mouth of the pit. In addition, a glassy verdigris exists around the pit. Furthermore, the pit measures <1 mm in diameter, but is extremely deep. In our study, we reproduced moundless pits by field testing in an area where moundless pits have often occurred. Moundless pits were also generated in synthetic freshwater through the dissolution of some selected components in a laboratory test. This synthetic freshwater contained 40 ppm SiO_2_, 50 ppm SO_4_^2−^, 10 ppm Cl^−^, and 10 ppm HCO_3_^−^. Surface analysis of the moundless pit revealed that the mouths of the pits were covered with thin films that were mainly composed of silica.

## 1 Introduction

Copper is widely used as a tubing material because of its ease of application, its high thermal conductivity, and its favourable corrosion resistance. Copper tubing is considered to be an essential component for supporting a social infrastructure, wherein it is employed in the heat conduction pipes of air conditioning units and in both tap water and hot water feed lines in houses and buildings. Although copper is considered a corrosion-resistant material in freshwater environments, such as in the above applications, some corrosion-related incidents have been reported for copper tubes used in some types of freshwater environments. Indeed, a number of studies have been published concerning the pitting corrosion of copper in freshwater since the 1950s [1, 2, 3, 4, 5, 6, 7, 8, 9, 10, 11, 12, 13, 14].

In previous related studies, the pitting corrosion of copper in freshwater has been commonly classified into various categories. In the United States, the pitting corrosion of copper in freshwater has generally been classified into three main categories, namely types I, II, and III [10, 13]. Type I corrosion tends to occur in hard and cold water. It is said that the carbon film that exists inside the copper tube plays an important role in the initiation and propagation of such corrosion [1], wherein the patina coating on the corrosion pit is generally composed of malachite (CuCO_3_·Cu(OH)_2_). Type II corrosion takes place in hot water that contains a high concentration of sulfate ions relative to bicarbonate ions. In this case, the narrower and deeper pits formed during type II corrosion are covered by a patina of brochantite (CuSO_4_·3Cu(OH)_2_). Type III pitting is caused by soft water and has a wide and shallow pit. In contrast, in Japan, only type I and type II classifications tend to exist [8], although the classification conditions are slightly different. More specifically, in Japan, type I pitting occurs under hard and cold water conditions, especially in groundwater containing a high free carbon dioxide content. However, type II pitting is classified as occurring both in soft and hot water, which contrasts to the United States classification system. The ratio proposed by Mattsson, namely the HCO_3_^−^/SO_4_^2−^ ratio, constitutes an index that distinguishes type I from type II corrosion, wherein type I occurs in the case where HCO_3_^−^/SO_4_^2−^ > 1, and type II occurs in the case where HCO_3_^−^/SO_4_^2−^ < 1 [4]. As in the case of the United States classification, in Japan, type I and type II pits also contain patinas composed of malachite and brochantite, respectively. In general, Japanese river waters are classified as soft water, since they contain comparatively low levels of Ca and Mg, and as a result, the majority of pitting damage detected in copper tubes in Japan was reported to be type II pitting. However, despite their different classifications, the morphological features of type I and II pitting are comparable, since both constitute a semispherical pit grown beneath a mound of verdigris [15, 16].

Interestingly, since the 1990s, pitting corrosion that is not classified as either type I or type II has been discovered in some areas of Japan, and such pitting corrosion has recently been discovered throughout Japan. This new type of pitting corrosion is known as “moundless” pitting corrosion due to its morphological characteristics [17]. Thus, we herein present and discuss the morphological aspects and water quality features of moundless pitting corrosion after field surveys and field tests in Noboribetsu City, Hokkaido Prefecture. In addition, a laboratory experiment is conducted using synthetic freshwater to reproduce the moundless pitting corrosion. The various corroded copper tube specimens are then examined by a range of analytical techniques, including X-ray diffraction (XRD), energy dispersive spectroscopy (EDS), Fourier transform infrared (FT-IR) spectroscopy, optical microscopy, and electron probe microanalysis (EPMA) in conjunction with scanning electron microscopy (SEM).

## 2 Materials and methods

### 2.1 Field surveys

**Figure 1:**
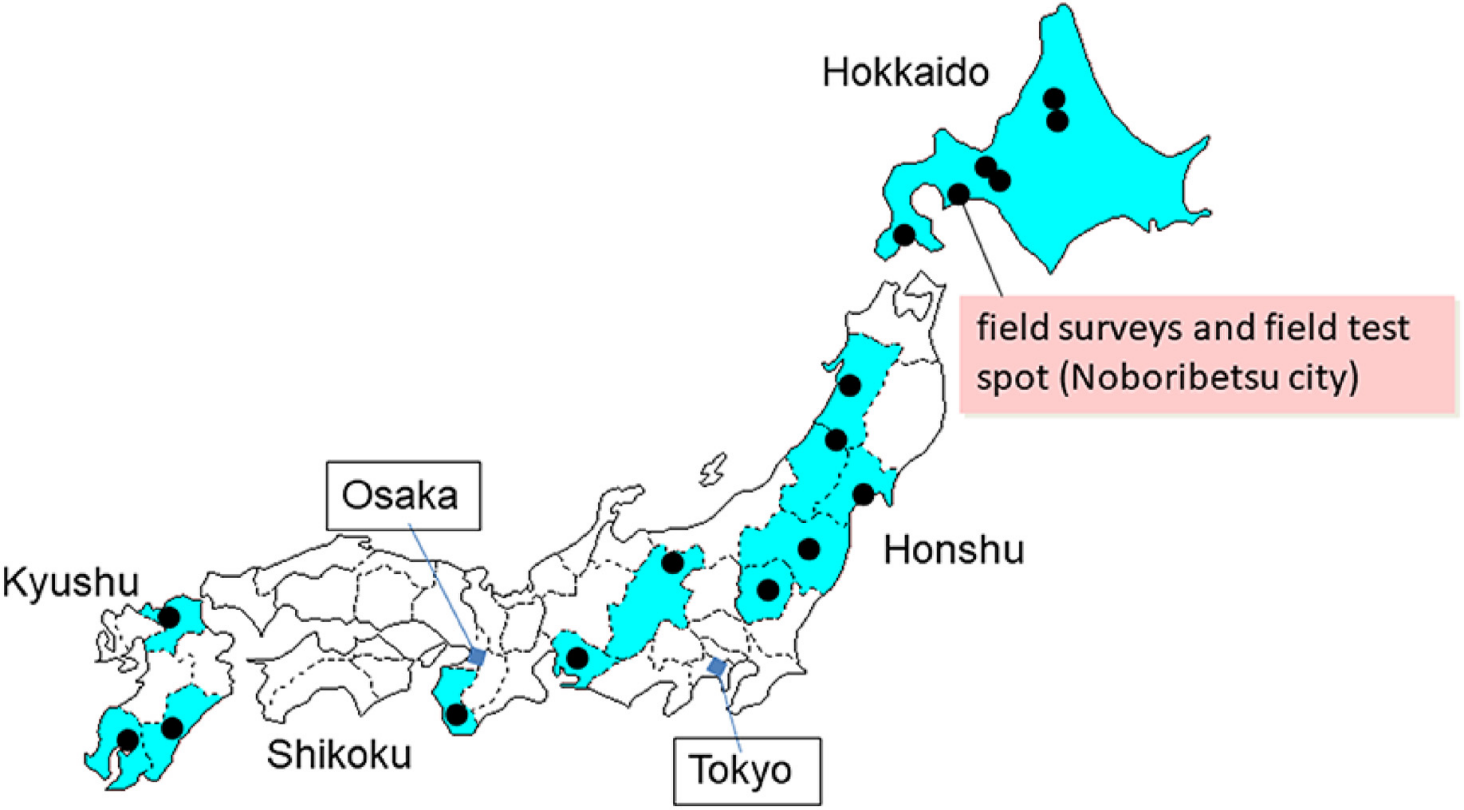
Locations of water leakage accidents caused by the moundless pitting corrosion of copper tubes in Japan.

Figure 1 shows the locations of water leakage accidents caused by the moundless pit corrosion of copper tubes in Japan. Noboribetsu City in the Hokkaido Prefecture was selected as our field survey location due to the fact that many such water leakage accidents were reported in this area, and also because our university is located in Muroran City, which is close to Noboribetsu City. The copper tube sections examined in this study were provided by homeowners who suffered from pinhole leak accidents that required replacement of the piping with new copper tubes. After transport to our laboratory, the tubes were cut axially using a band saw, and the inside of each tube was observed under an optical microscope (Nikon SMZ1500). The verdigris around the pit was analysed using XRD (Bruker D8 Discover) and FT-IR (Jasco FT/IR 4000). Analyses of the tap water samples supplied from water treatment plants in Noboribetsu City were requested once per month for a period of one year, wherein the pH, electrical conductivity, hardness, sulfate ion concentration, chloride ion concentration, bicarbonate ion concentration, and silica content were evaluated.

### 2.2 Field tests

**Figure 2:**
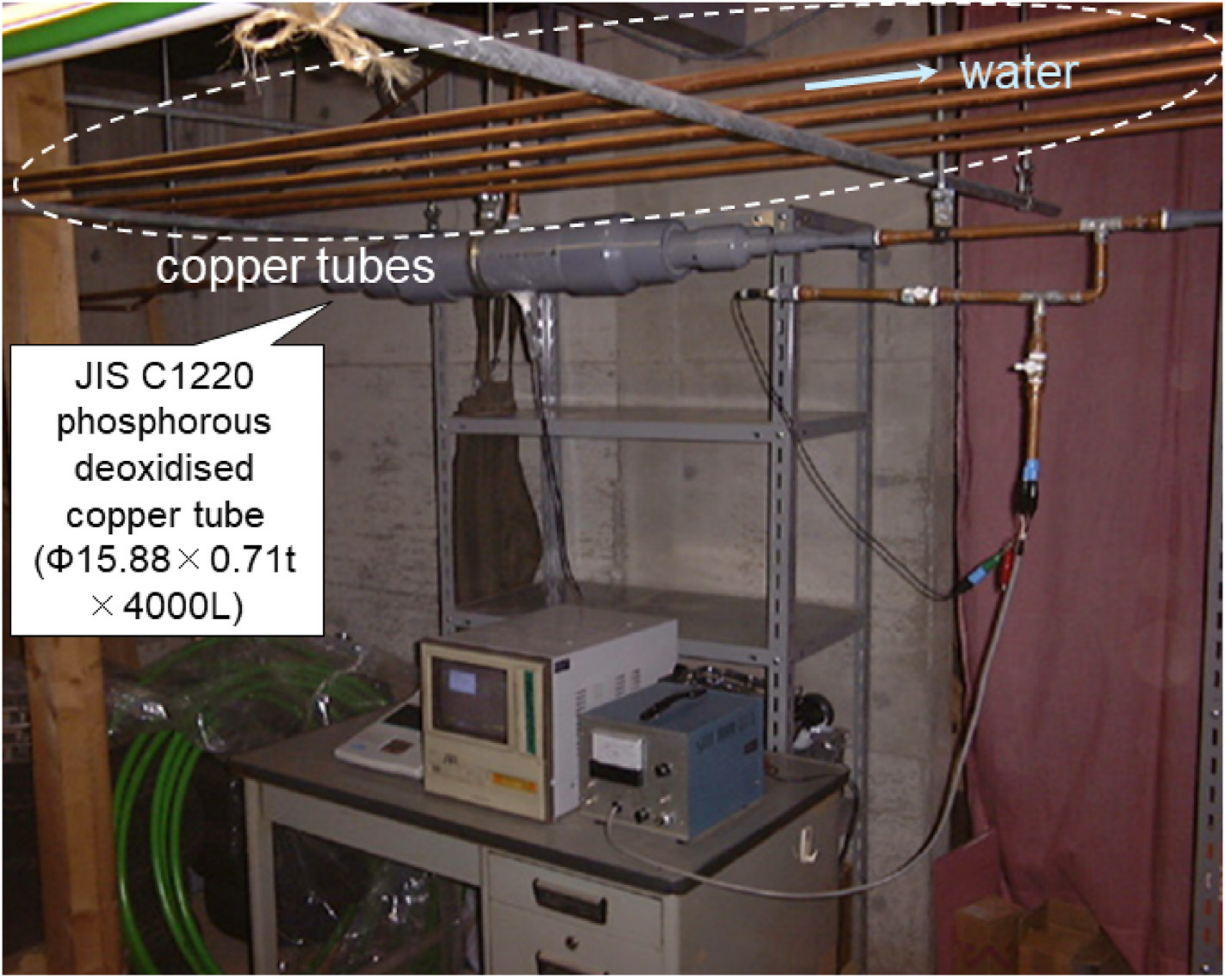
Appearance of field test system installed in the basement of a local plumbing contractor. Five copper tubes measuring 4000 mm in length were connected with brazing joints, and the water inside the tubes was replaced once per week.

Figure 2 shows a field test centre installed in the basement of a local plumbing contractor. Five copper tubes composed of JIS H3300 C1220 phosphorus-deoxidised copper and measuring 15.88 mm in diameter, 0.71 mm in thickness, and 4000 mm in length, were used. These five copper tubes were connected in series using brazing joints and installed parallel to the ground. Tap water was introduced into tubes at full water level and was allowed to remain stagnant, being replaced once per week by opening a hand-valve. All tubes were removed from the installation four years later and cut axially with a band saw to separate into the top (ceiling) and bottom (ground) sections. The insides of these tube segments were observed by optical microscopy and were analysed by EDS and EPMA (JEOL JXA-8900R).

### 2.3 Laboratory tests

Artificial freshwater was prepared to reproduce moundless pitting corrosion in the laboratory environment. We used synthetic freshwater to simulate the real tap water that caused such corrosion in actual systems. Since the presence of silica, in addition to sulfate, chloride, and bicarbonate ions, appears necessary for the generation of moundless pits, CaSO_4_·2H_2_O (a sulfate ion source), NaCl (a chloride ion source), and NaHCO_3_ (a bicarbonate ion source) were added to the ion-exchanged water. Although silica is often injected in the form of water-soluble Na_2_SiO_3_, such water is known to become highly basic, and so to return the pH back to a neutral value of 7, an acid must be added. To avoid this complicated protocol, amorphous SiO_2_ was used as the silica source for the test water. Although the dissolution of SiO_2_ in water is slow at ambient temperature, taking ∼3 weeks to reach equilibrium [18], it is known that the solubility of SiO_2_ in water increases with increasing temperature [19]. Thus, we injected a considerable amount of amorphous SiO_2_ into high-temperature water, and then filtered the final water sample to remove any undissolved SiO_2_. The final levels of silica, sulfate ions, chloride ions, and bicarbonate ions in the synthetic freshwater were 40, 50, 10, and 10 ppm, respectively. The pH of this synthetic freshwater was 7, and the electrical conductivity was 17 mS/m, which is comparable to that of tap water obtained from a moundless pitting area (i.e., pH 6.8; electrical conductivity = 18 mS/m). The synthetic freshwater (500 mL) was stored in a polypropylene container. Longitudinally-halved phosphorus-deoxidised copper tubes measuring 100 mm in length were covered with silicone resin, with the exception of an exposed inner area of 8.8 cm^2^. Each copper tube specimen was immersed in synthetic freshwater prepared at ambient temperature under stagnant conditions for 1 year. During the 1-year immersion period, the test water was never refreshed, and at the end of the immersion period, each specimen was removed from the test water and allowed to dry naturally. After natural drying, the exposed area was observed by optical microscopy and analysed using EPMA.

## 3 Results and discussion

### 3.1 Field surveys

**Figure 3:**
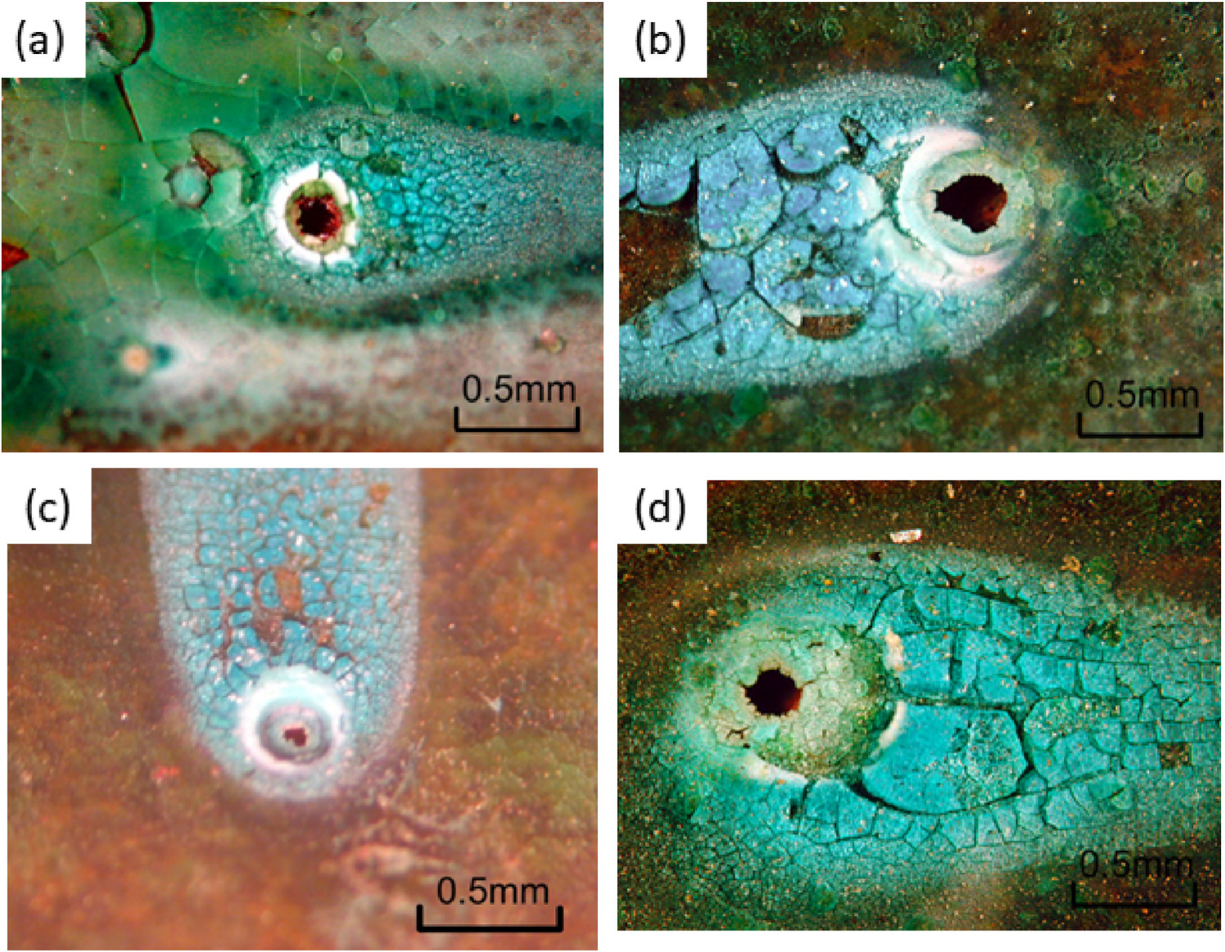
The new type of moundless pitting corrosion detected in domestic water copper feed tubes removed from houses a, b, c and d in the Noboribetsu area of the Hokkaido Prefecture.

**Figure 4:**
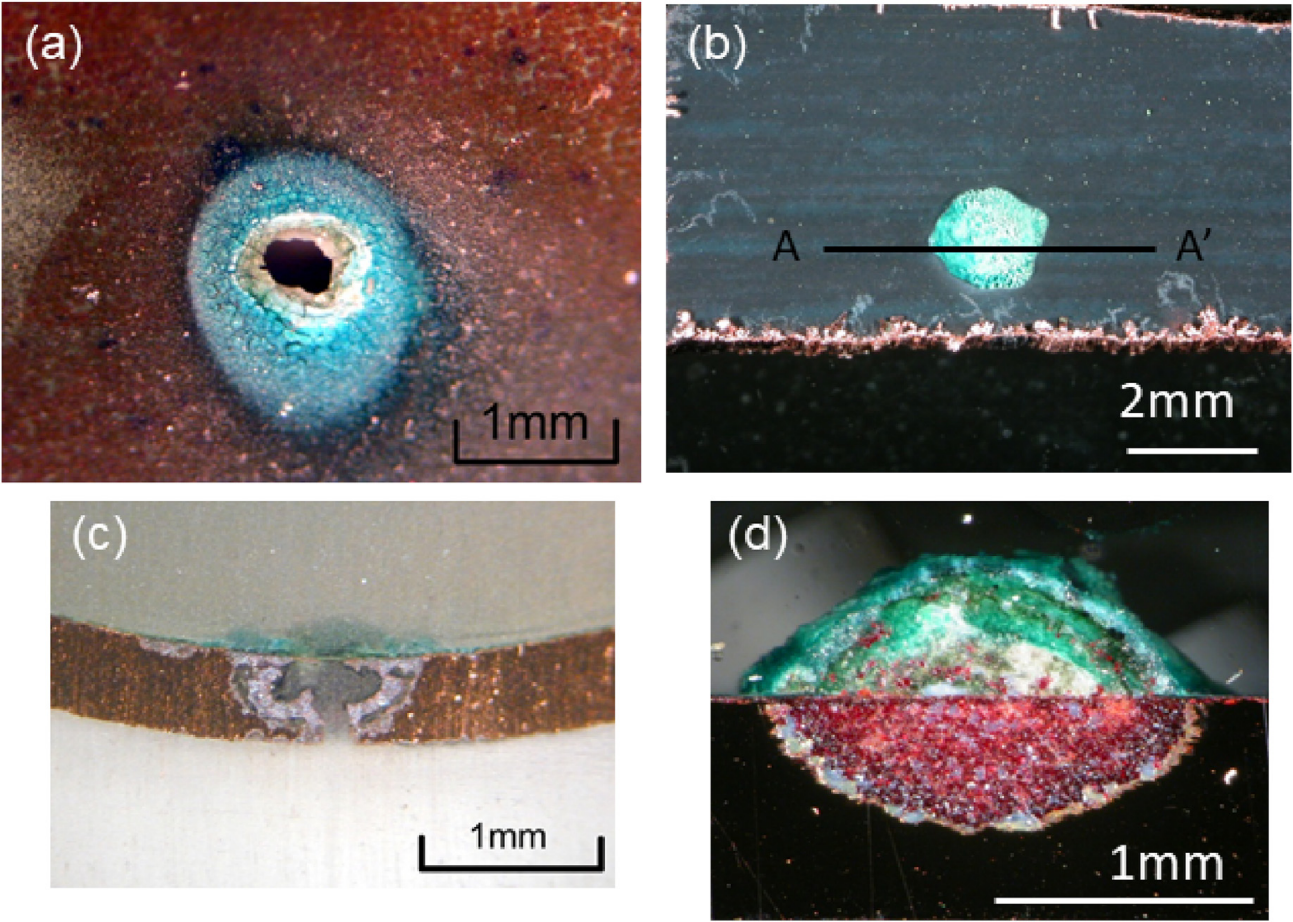
(a) An image of moundless pitting corrosion and (c) its cross-section. (b) An image of type II pitting corrosion and (d) its cross-section.

Figure 3 shows the new type of pitting corrosion that occurred on the inner surfaces of the copper tubes employed in the water distribution system of Noboribetsu City. These pictures are parts of pits caused 255 water leakage accidents in Noboribetsu City. This type of pit caused almost all of 255 water leakage accidents. The most distinctive feature of these pits is that the pit mouths are generally open and are not covered with a mound of verdigris; as shown in Figure 3, the ring of the verdigris deposit surrounds the pit mouth. The reflection of light from this verdigris around the pit mouth indicates that the deposited verdigris is vitrified. In addition, Figure 4 shows the new type of pitting corrosion in addition to type II pitting corrosion for comparison purposes. More specifically, the type II pit possesses a mound of verdigris, and the pit propagates under the mound, whereas the new pit type has no mound covering its pit mouth. This new type of pitting corrosion was therefore named “moundless” pitting corrosion because of its morphological features. Another characteristic feature of this moundless pit is its comparatively small pit diameter, which typically measures <1 mm; the pits of type I and type II pits generally possess diameters of a few millimetres. Furthermore, the preferential downward growth orientation can be seen clearly in the cross-sectional view shown in Figure 4(a).

**Figure 5:**
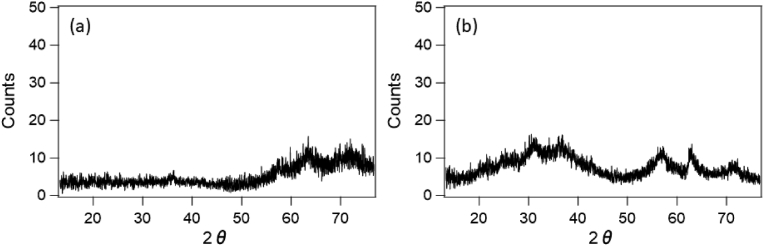
XRD patterns of the verdigris collected from around the moundless pit mouth of houses a and b.

**Figure 6:**
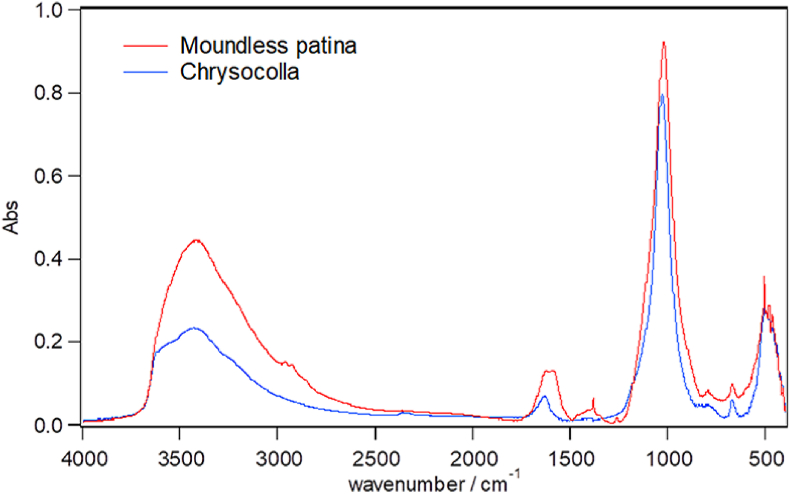
FT-IR spectrum of the verdigris around the moundless pit mouth. The corresponding spectrum of chrysocolla is also provided for comparison.

Figure 5 shows XRD pattern of the verdigris around the pit mouth, wherein the broad pattern and lack of any specific peaks indicates that this verdigris is amorphous. In addition, Figure 6 shows the FT-IR pattern of the verdigris along with that of the amorphous copper-containing silicate mineral chrysocolla (Cu_2_H_2_Si_2_O_5_(OH)_4_·nH_2_O). Upon comparison, it appears that the verdigris of around the pit mouth is composed of chrysocolla.

**Figure 7:**
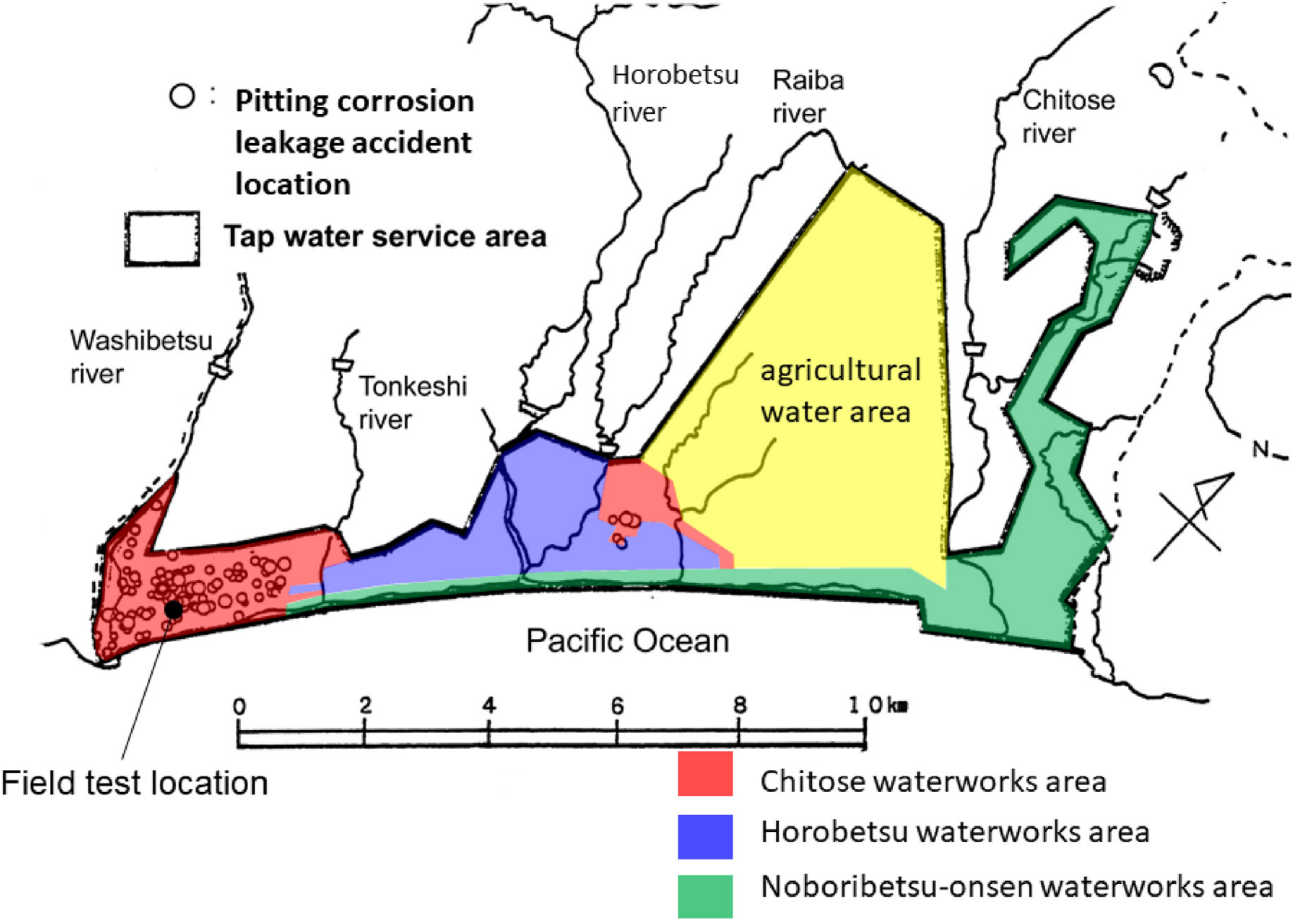
Map of the tap water service area and the locations of water leakage accidents caused by moundless pitting corrosion in Noboribetsu City. The tap water in Noboribetsu City is distributed from three water treatment plants, namely the Chitose, Horobetsu and Noboribetsu onsen plants.

Figure 7 shows an outline of the tap water service area in Noboribetsu City along with the locations of the water leakage accidents caused by moundless pitting corrosion. At the time of this investigation, the tap water provided to Noboribetsu City was distributed from three waterworks, namely Chitose, Horobetsu, and Noboribetsu-onsen. As can be seen from the figure, the main concentration of leakage accidents correlated with the Chitose waterworks area, with no such accidents being reported in the Horobetsu and Noboribetsu-onsen waterworks area. These results therefore suggest that the water quality is a key factor in terms of the initiation and propagation of moundless pitting corrosion in Noboribetsu City.

**Figure 8:**
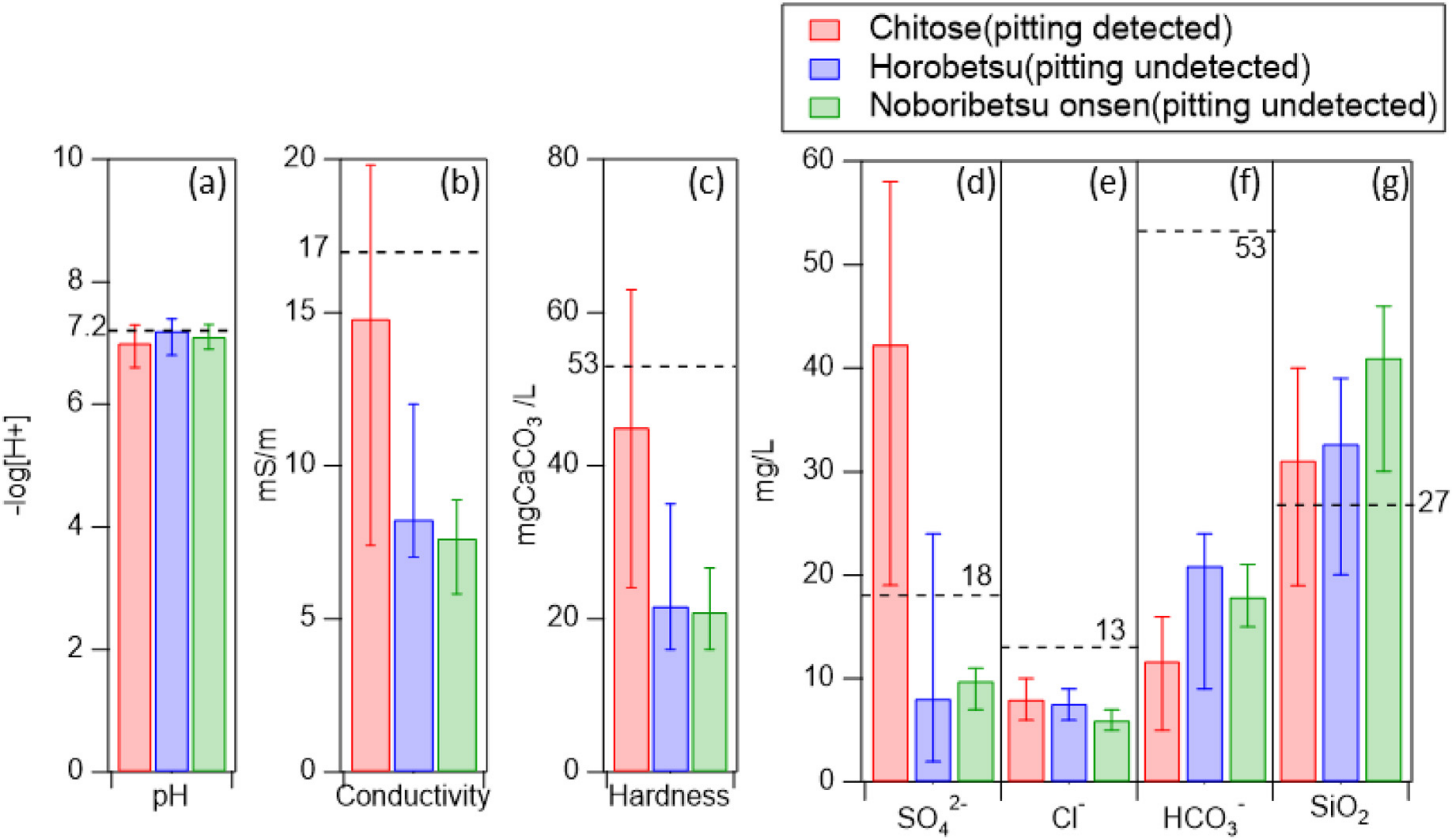
Water quality factors of the three water systems in the Noboribetsu area of the Hokkaido Prefecture (horizontal broken lines refer to the average levels for Japanese tap water). (a) pH, (b) electric conductivity, (c) hardness, (d) sulfate ion concentration, (e) chloride ion concentration, (f) bicarbonate ion concentration, (g) silica content.

Figure 8 shows a comparison of the water qualities among the three water supply systems in Noboribetsu City, wherein the average level of each factor is also indicated, as determined from the database for Japanese tap water [20]. As indicated by the data presented in Figure 8, a silica level of 30–40 ppm was observed for all three water systems, which is higher than the average Japanese level (27 ppm). However, the silica level in the Chitose water system was 10 ppm lower than that in the Noboribetsu-onsen water system, where no moundless pitting accidents were reported. Thus, although a high silica level might be one of the factors correlated with moundless pitting, it is certainly not a decisive factor for its initiation. It is also noteworthy that the bicarbonate ion concentration is lower than the Japanese average level of 53 ppm for all the three Noboribetsu water systems. It has been claimed that bicarbonate possesses a pH buffering effect, and could therefore decelerate pitting propagation by minimising the pH shift of the water retained in the pit [8, 16]. Thus, pitting propagation might proceed more rapidly in water with a comparatively low bicarbonate level. A high concentration of chloride ions is also believed to be detrimental in terms of the copper pipe stability to pitting. However, in all cases, the chloride ion levels were <10 ppm, which is lower than the Japanese average of 13 ppm. Indeed, the differences in levels of silica, bicarbonate ions, and chloride ions among the three Noboribetsu water systems were not significant, and so it was considered that the sulfate ion may be a key factor in determining the degree to which moundless corrosion took place the various water systems examined herein. Indeed, the Chitose water sample was found to contain a high sulfate ion concentration of 42 ppm, which is five times higher than those of the Horobetsu and the Noboribetsu-onsen water systems, and twice as high as the Japanese average. High sulfate ion in water promotes the pit propagation rate [21]. It can also be seen from Figure 8 that the electrical conductivity and hardness of the Chitose water were approximately twice as high as those of the Horobetsu and Noboribetsu-onsen water samples. It was therefore considered that moundless pitting in Noboribetsu City was promoted by a comparatively high silica concentration (>30 ppm), a relatively high sulfate ion concentration (>40 ppm), and a low bicarbonate ion concentration (<10 ppm).

### 3.2 Field tests

**Figure 9:**
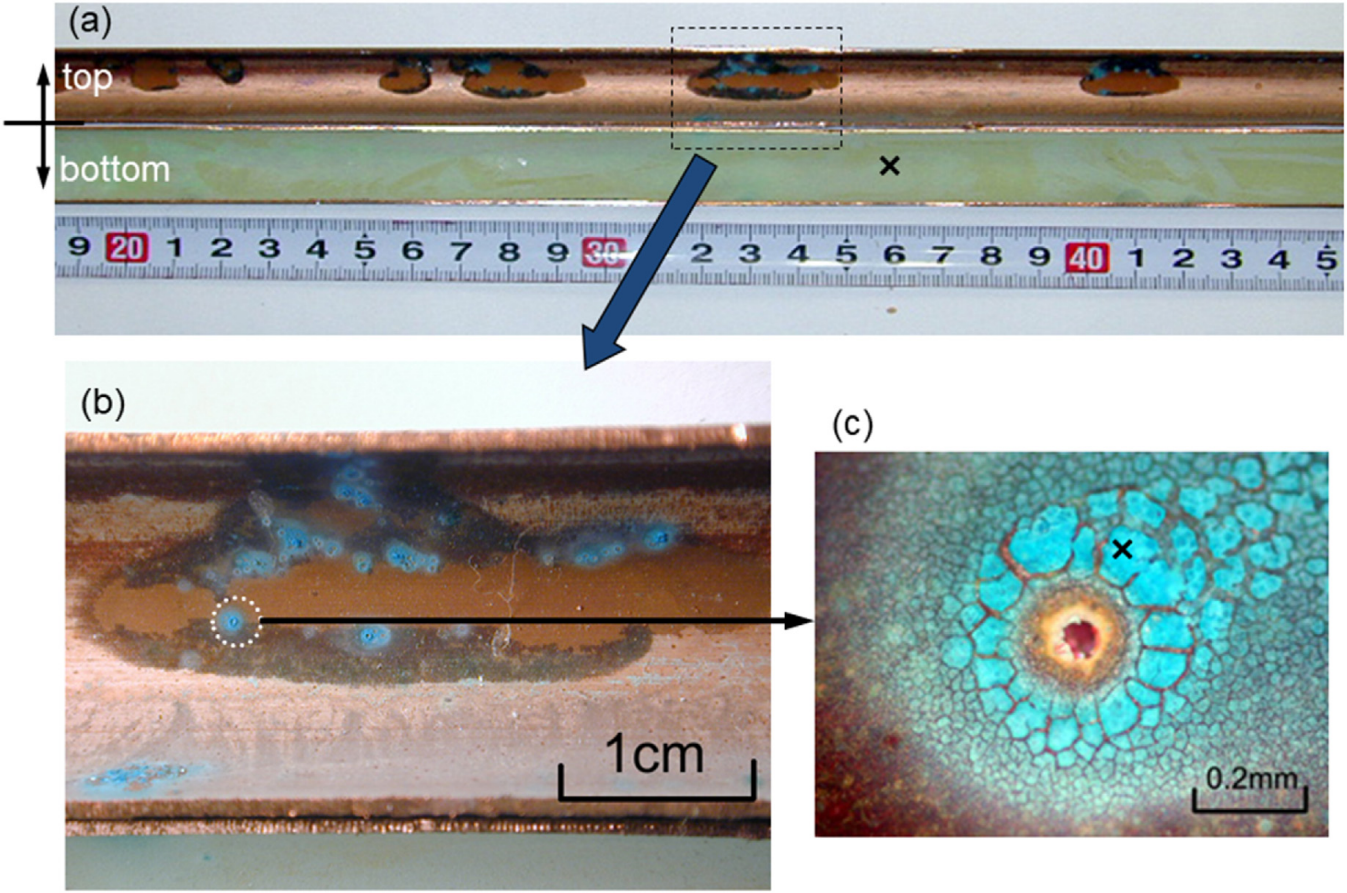
Inner surface of a copper tube after the 4-year field exposure test in the Noboribetsu area. The emergence of moundless pitting corrosion can be clearly observed: (a) General view; (b) Enlarged view of the selected area of Fig.9a; (c) Enlarged view of the selected area of Fig.9b. x-marks are analysis points for EDS.

**Figure 10:**
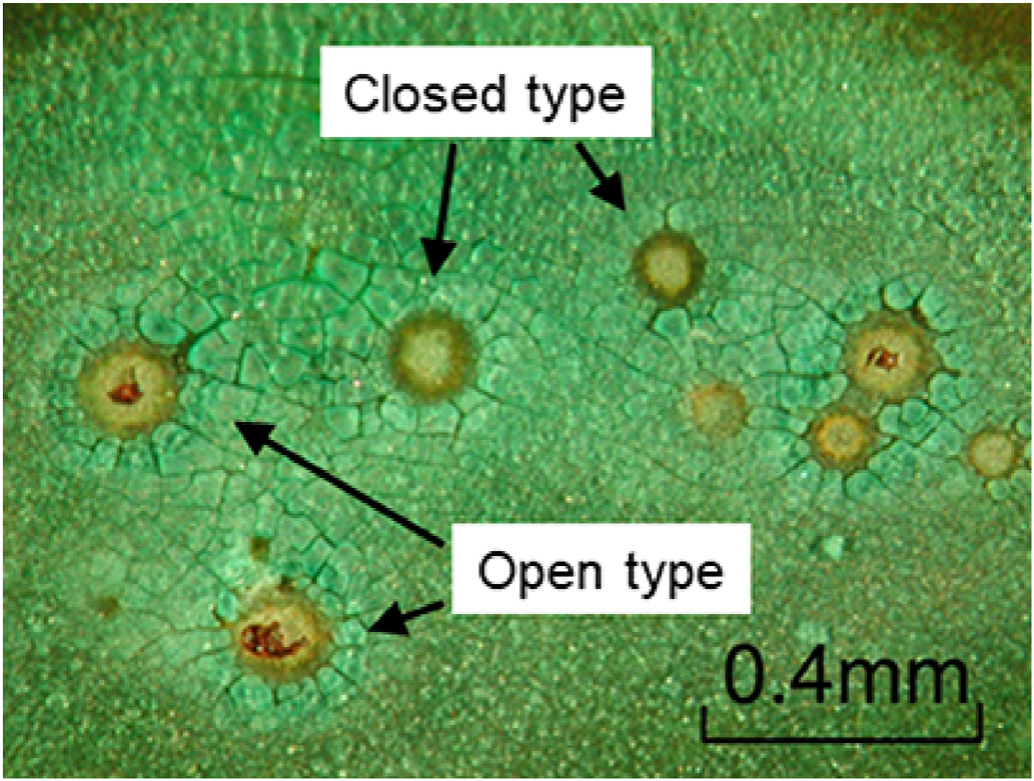
Initial stage of development of moundless pits wherein closed-mouth pits can be observed.

Figure 9(a) shows the inner surface of a typical copper tube after the 4-year test, wherein the top (ceiling) and bottom (ground) sections of the copper surface are clearly distinguishable from one another. More specifically, the bottom section is homogeneously covered with a greenish-white scale, while islands showing discoloured brown spots can be seen on the top section of the tube. In addition, as shown in Figure 9(b), verdigris was detected along the boundaries of the brown spots, and many open-mouth pits were observed (see Figure 9(c)), which were surrounded by a glassy verdigris. Thus, using this field simulation test, the moundless pits detected in the water system were successfully reproduced, although the dimensions of the reproduced pits in this field test were smaller than those obtained in the field survey. A detailed inspection of the test piece showed that not all pits formed during the field test were open pits (Figure 10), wherein some of the closed pits possessed mouths covered with a film that could be broken using a needle. We therefore considered that in the initial stage of moundless pit development, the pit mouth is closed and covered with a thin film. During pit growth (or during sample drying), this film is broken by the impact of flowing water to give an open-mouth morphology.

**Figure 11:**
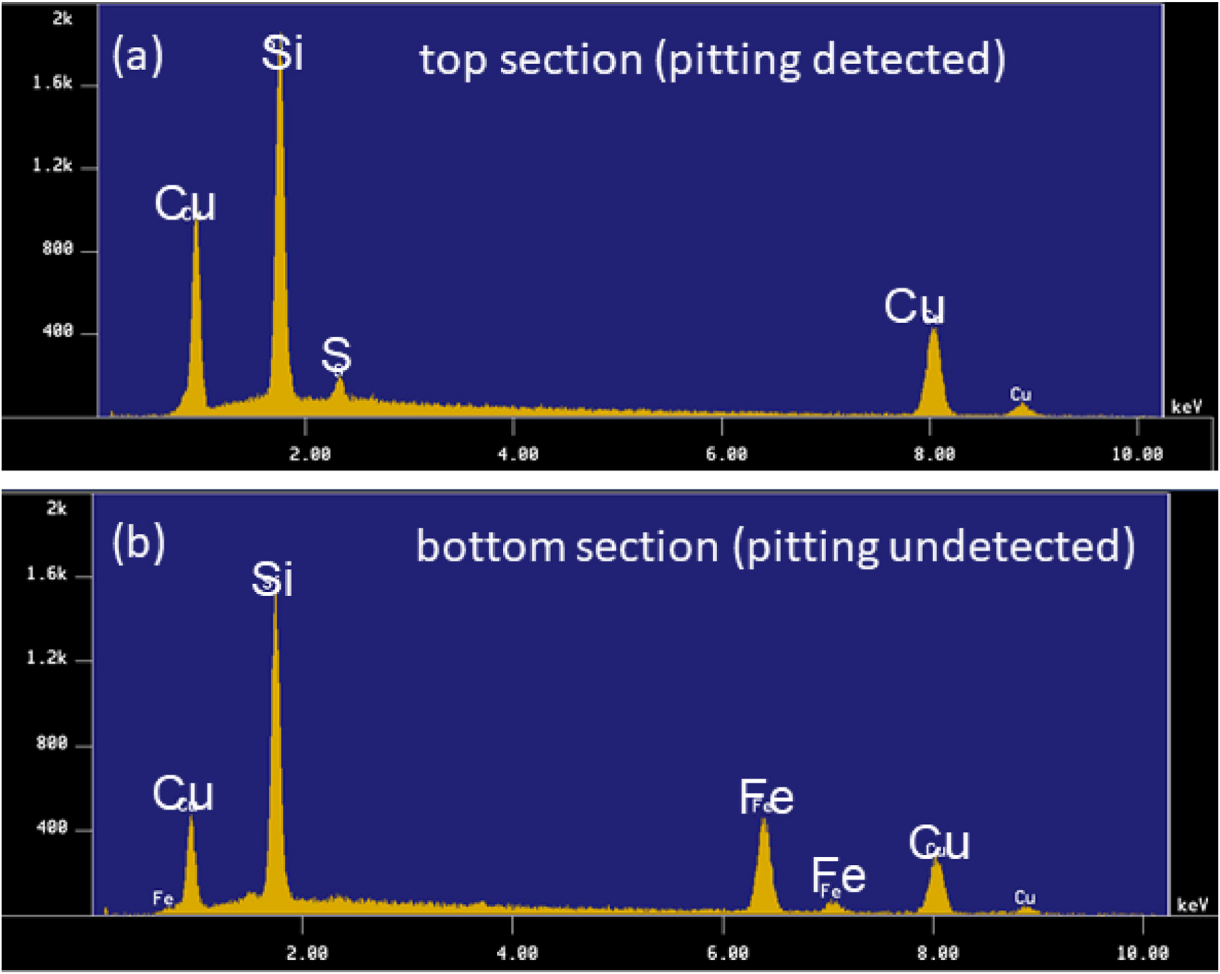
EDS spectra of the tube specimens shown in Figure 9, i.e., (a) the top (ceiling) section where pit formation was observed, and (b) the bottom (floor) section of the pit where the greenish white scale was observed.

**Figure 12:**
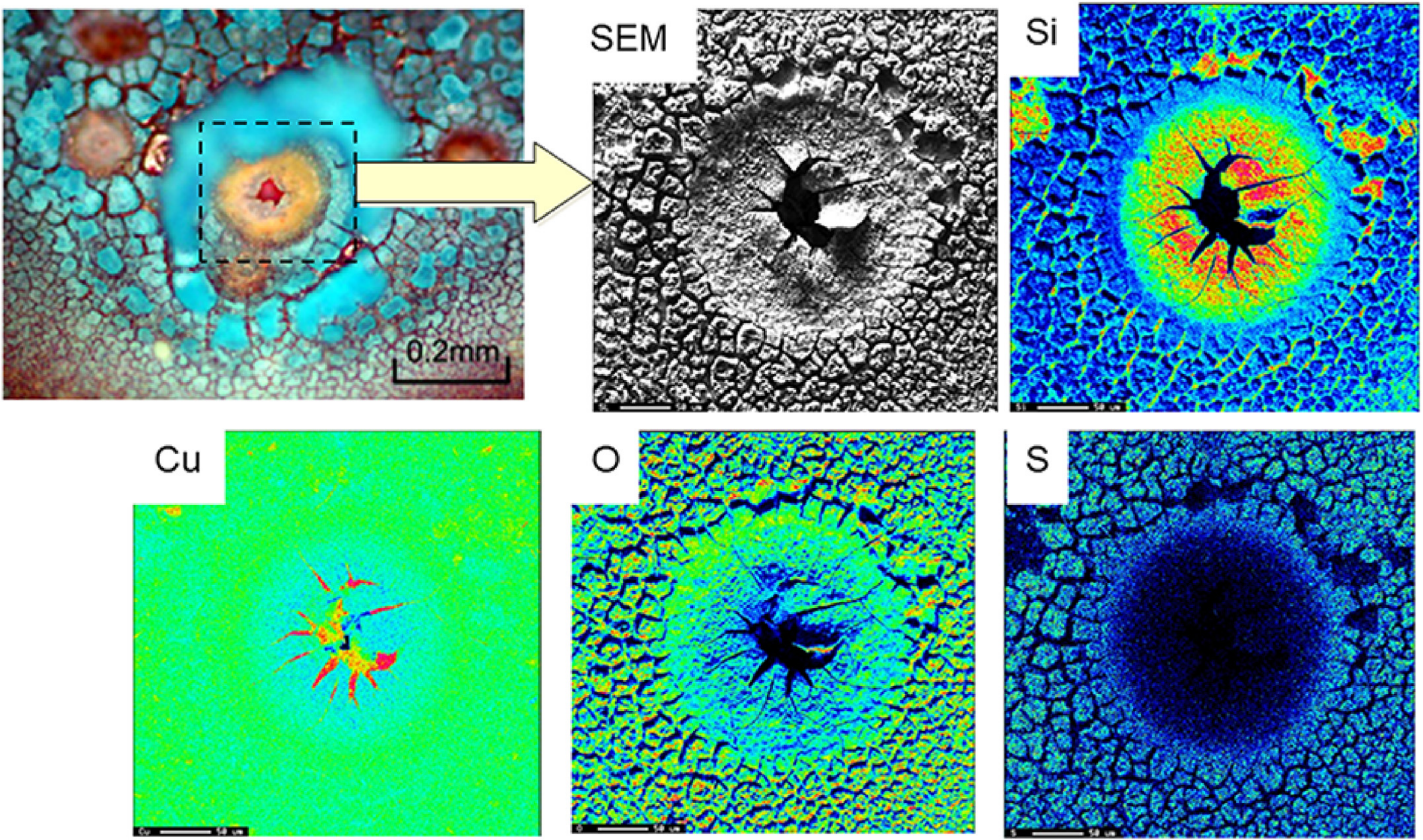
EPMA mapping of the elements present around a moundless pit. The elements analysed are Si, Cu, O, and S.

We then performed EDS on both the tube sample containing moundless pits (i.e., the top component of the tube shown in Figure 9(a)) and the tube sample bearing a greenish white scale (i.e., the bottom component of the tube shown in Figure 9(a)). As presented in Figure 11, for the top (ceiling) region of the copper tube, Cu, Si, and S were detected by EDS analysis, whereas Fe, Cu, and Si were detected in the bottom (floor) region. The detected Fe represents the corrosion product of iron arising from the ductile cast iron pipe used in the tap water transportation line from the waterworks to each houses. This result indicates that the greenish white scale formed by the reaction of Fe and Si in the water, and its subsequent deposition on the inner bottom surface of the copper tube. This scale of compound made from Fe and Si protected the copper surface, and so no moundless pits were formed on the bottom surface. Figure 12 shows typical EPMA maps for the elemental distribution profiles of the scale surrounding the moundless pit during the field test. More specifically, the condensation of Si was confirmed, and so we speculated that the scale film was formed from a silica component present in water. We suspected that the formation of this silica film was one of the key factors in initiating formation of the moundless pit.

### 3.3 Laboratory tests

**Figure 13:**
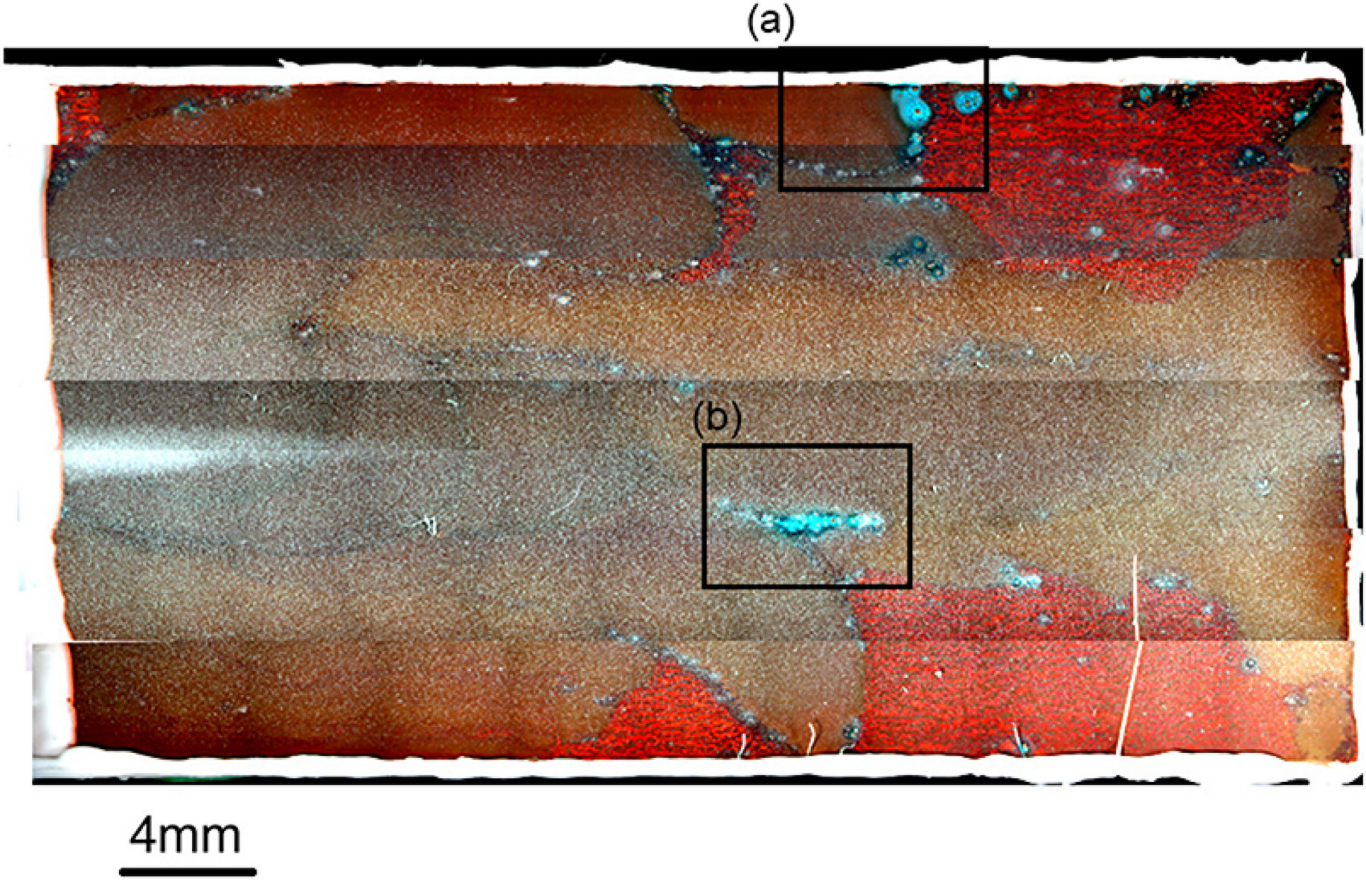
The inner surface of a copper tube specimen after 1 year of immersion in synthetic freshwater during the laboratory test. Areas (a) and (b) are enlarged in Figure 14.

**Figure 14:**
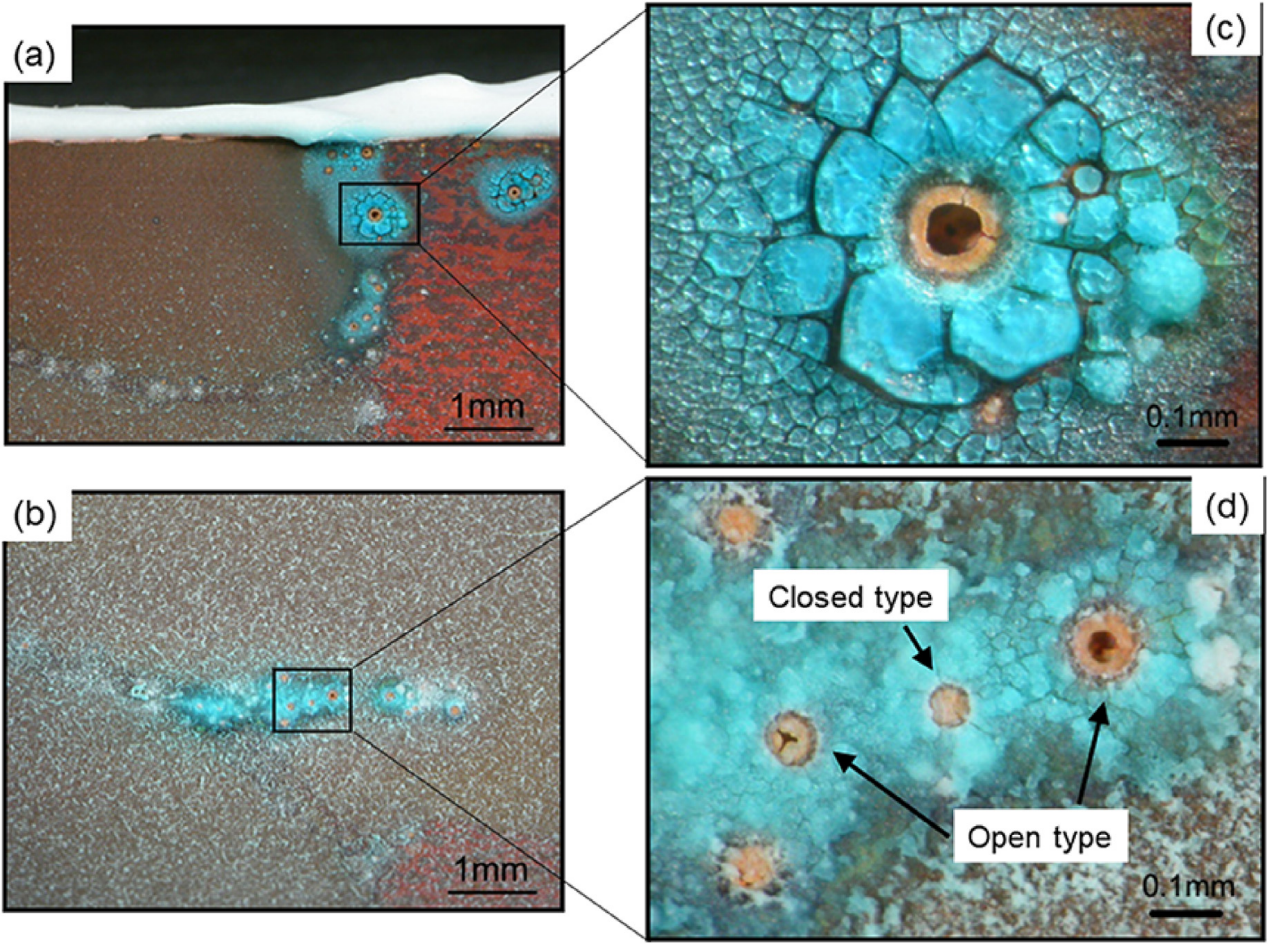
(a, b) Optical micrographs of the areas indicated in Figure 13(a) and 13(b) showing the reproduction of moundless pitting corrosion during a laboratory test using synthetic freshwater (c, d) Magnified images of the verdigris corresponding to these areas.

**Figure 15:**
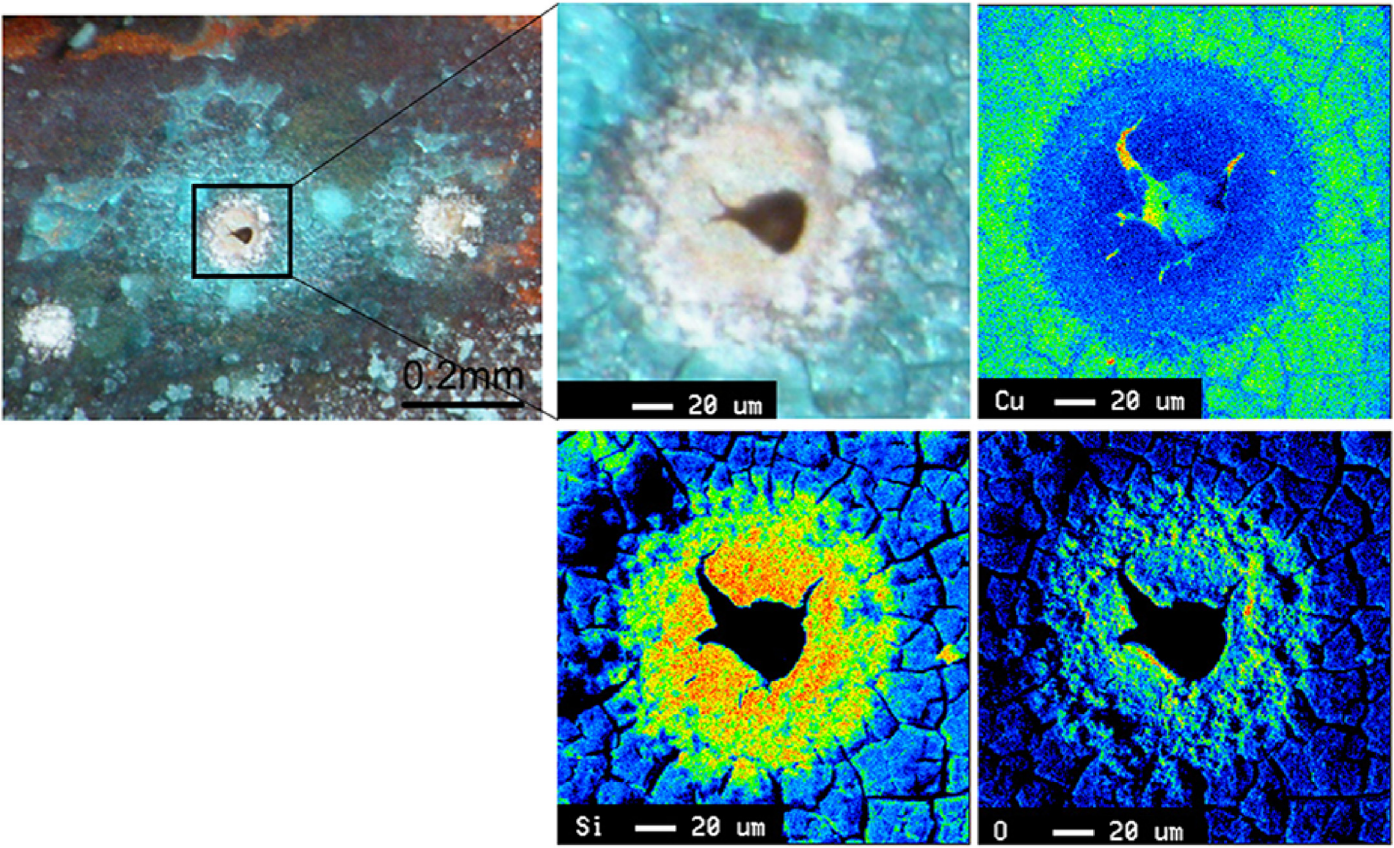
EPMA mapping of the elemental distributions around a moundless pit formed during the laboratory test using synthetic freshwater. The elements analysed are Cu, Si and O.

Figure 13 shows the appearance of the entire surface of the copper tube test piece after 1 year. As can be seen from the figure, verdigris spots were discretely visible over the discoloured brown surface. In addition, Figure 14(a) and 14(b) show the optical micrographs of the areas indicated in Figure 13(a) and 13(b), whereas Figure 14(c) and 14(d) show magnified images of the verdigris corresponding to these areas. In some cases, the pits were open and a glassy verdigris was observed around the pit mouth; this reproduces the characteristic features of the moundless pits detected in the field. Pits with completely closed mouths were also detected (Figure 14(d)), as detected in the field (Figure 10). Furthermore, Figure 15 shows the EPMA mapping images of a moundless pit reproduced in the laboratory tests, wherein the presence of Si was clearly confirmed. This result is consistent with the field test results presented in Figure 12, thereby indicating that moundless pit formation was observed in the synthetic freshwater as well as in the field test using tap water distributed from a Chitose water treatment plant. It was therefore confirmed that the formation of moundless pits was initiated in freshwater containing silica, sulfate ions, chloride ions, and bicarbonate ions. The depth of the pit shown in Figure 14(c) is the deepest of 0.216 mm, which corresponds to a corrosion rate of 0.216 mm/y, which is considered to be a relatively high rate.

**Figure 16:**
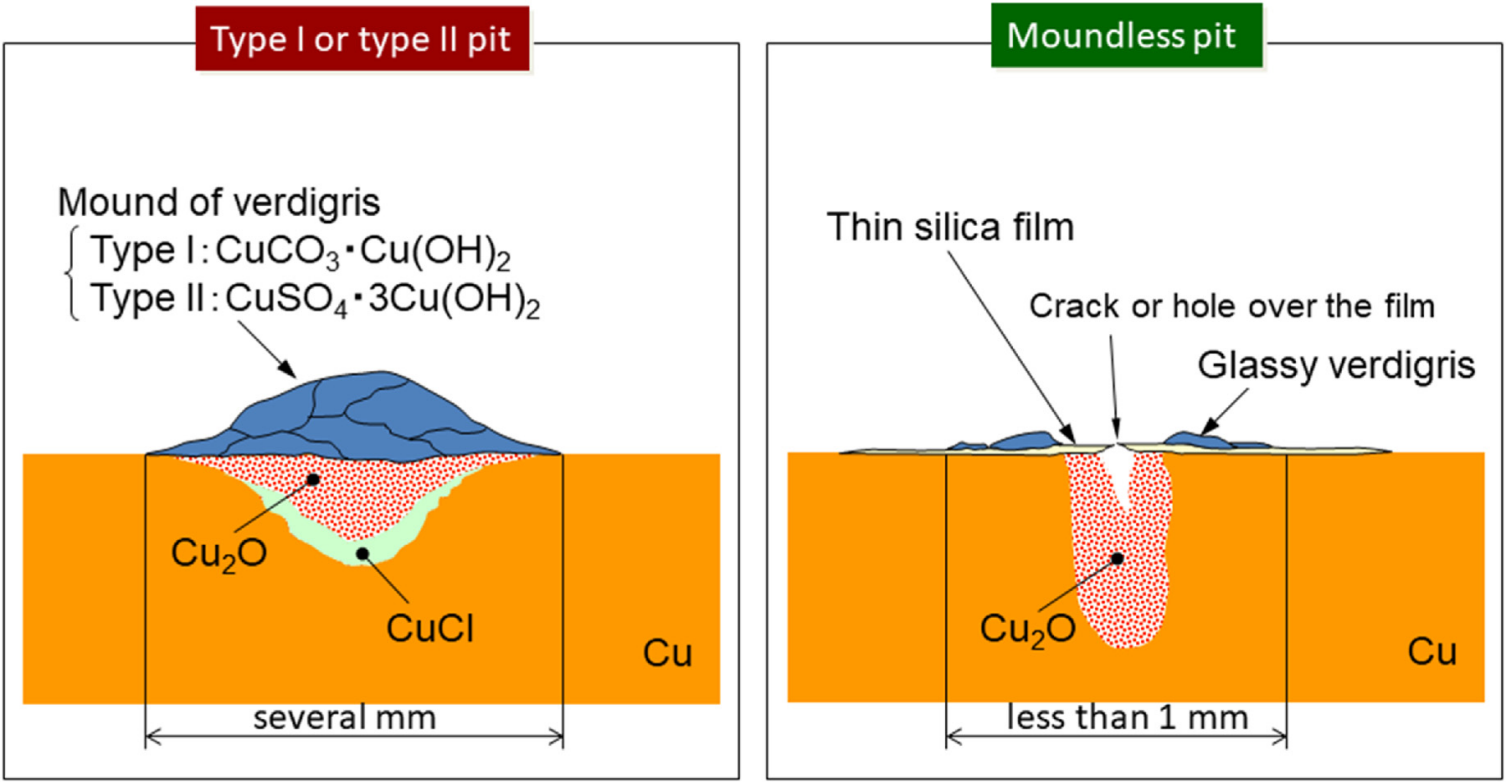
Schematic representation of the type I and type II pits (left-hand panel) and that of a moundless pit (right-hand panel).

From the results of the field and laboratory tests, the constitution of the moundless pit was estimated, as shown in the right-hand panel of Figure 16. The left-hand panel of Figure 16 gives the constitutions of the conventional type I and II pits for comparison [3, 16]. The presence of verdigris covering the pit top is essential for pit growth in type I or type II pits. This verdigris functions as a shield for the water retained in the pit against exchange with the surrounding water, thereby promoting a reduction in the pH of the water in the pit. The reduced pH of the solution in the pit activates an anodic reaction and thereby promotes the resultant pitting propagation. Since there was no verdigris at the top of the moundless pits, I thought that some other process, distinct from the process of initiation and propagation of type I and type II pitting, was necessary to explain the moundless pit. However, as shown in Figure 10 and Figure 14(b), I discovered that closed pits were present together with the open pits. At least in the early stage of development of moundless pit, therefore, the pit top was not entirely open and was covered with thin surface film. This film may have acted as a shield between the solution in the pit and the surrounding environmental water, much as verdigris does in the case of type I and type II pits. Since the film thin and fragile, it was lost after a certain amount of propagation of the pitting or even after the pipe sampling after test, thus yielding the moundless morphology of the concerned pits. In light of this, we now believe that the thin film at the pit top was essential for the development of the moundless pits. Judging from the EPMA analysis (Figure12 and Figure 15), this film was composed mainly of Si. I do not as yet know the detailed mechanism of formation of the thin film, but I am investigating it.

## 4 Conclusions

A new type of pitting corrosion, namely moundless pitting corrosion, which has recently been reported many times in Japan, was investigated by field surveys, field tests, and laboratory simulations. It was found that the mouths of these new pits are open instead of being covered with a mound of verdigris, as is commonly observed for conventional type I and type II pits. In addition, a glassy verdigris, which is composed of an amorphous copper-containing silicate mineral, was found to exist around the mouths of these pits. Upon examination of the water quality of various regions of the Noboribetsu City in the Hokkaido Prefecture to determine the prerequisites for moundless pit formation, it was found that overall, the silica level was slightly higher than the average Japanese level. In addition, the levels of sulfate ions in pitting detected area were also higher than the Japanese average, while those of bicarbonate ions were lower than the Japanese average; however, differences existed between the water regions. Furthermore, we succeeded in reproducing moundless pits in Noboribetsu city using a 4-year field test. Following this simulation, we found that the mouths of some pits were closed in the early stages, and that these pits were covered with thin films that contained mainly silica. We also succeeded in reproducing moundless pits using artificial freshwater during a 1-year laboratory test using artificial freshwater containing 40 ppm silica, 50 ppm sulfate ions, 10 ppm chloride ions, and 10 ppm bicarbonate ions. It was therefore concluded that the formation of moundless pits is largely dependent on the water quality, and silica is considered indispensable for their generation.

## Declarations

### Author contribution statement

Masahiro Sakai: Conceived and designed the experiments; Performed the experiments; Analyzed and interpreted the data; Contributed reagents, materials, analysis tools or data; Wrote the paper.

### Funding statement

This work was supported by the Education Ministry of Japan under grant number 19760500.

### Data availability statement

Data associated with this study has been deposited at Muroran Institute of technology.

### Declaration of interests statement

The authors declare no conflict of interest.

### Additional information

No additional information is available for this paper.
